# *USH2A* gene variants cause Keratoconus and Usher syndrome phenotypes in Pakistani families

**DOI:** 10.1186/s12886-021-01957-9

**Published:** 2021-04-29

**Authors:** Asif Naveed Ahmed, Raheel Tahir, Niamat Khan, Mushtaq Ahmad, Muhammad Dawood, Abdul Basit, Muhammad Yasin, Maha Nowshid, Muhammad Marwan, Komal Sultan, Shamim Saleha

**Affiliations:** 1 Department of Biotechnology and Genetic Engineering, Kohat University of Science and Technology (KUST), Kohat, 26000 Khyber Pakhtunkhwa Pakistan; 2 Medical Teaching Institution, Hayatabad Medical Complex, Peshawar, 25000 Khyber Pakhtunkhwa Pakistan

**Keywords:** Recessive RP, USH2, Pakistani families, *USH2A* variants, Sanger sequencing

## Abstract

**Background:**

Retinitis pigmentosa (RP) is the most common inherited retinal dystrophy, affecting approximately 1 in 4000 individuals worldwide. The most common form of syndromic RP is Usher syndrome (USH) accounting for approximately 20–30 % of RP cases. Mutations in the *USH2A* gene cause a significant proportion of recessive non-syndromic RP and USH type II (USH2). This study aimed to determine the causative role of the *USH2A *gene in autosomal recessive inherited ocular diseases and to establish genotype-phenotype correlation associated with USH2A variants.

**Methods:**

We performed direct Sanger sequencing and co-segregation analysis of the *USH2A* gene to identify disease causing variants in a non-syndromic RP family, two USH2 families and two Keratoconus (KC) families.

**Results:**

Disease causing variants in the *USH2A* gene were identified in two families displayed KC and USH2 phenotypes. A novel variant c.4029T > G, p.Asn1343Lys in the *USH2A* gene was detected in a Pakistani family with KC phenotype. In addition, a missense variant (c.7334 C > T, p. Ser2445Phe) in the *USH2A* gene was found segregating in another Pakistani family with USH2 phenotype. Homozygosity of identified missense *USH2A* variants was found associated with autosomal recessive inherited KC and USH2 phenotypes in investigated families. These variants were not detected in ethnically matched healthy controls. Moreover, the *USH2A* variants were predicted to be deleterious or potentially disease causing by PolyPhen-2, PROVEAN and SIFT.

**Conclusions:**

This study provided first evidence for association of a novel *USH2A* variant with KC phenotype in a Pakistani family as well as established the phenotype-genotype correlation of a *USH2A *variant (c.7334 C > T, p. Ser2445Phe) with USH2 phenotype in another Pakistani family. The phenotype-genotype correlations established in present study may improve clinical diagnosis of affected individuals for better management and counseling.

## Background

Inherited retinal dystrophies (IRDs) are a broad group of clinically and genetically heterogeneous rare eye diseases resulting in progressive visual impairment or blindness [1]. Retinitis pigmentosa (RP; MIM#268,000) is the most prevalent form of IRDs, affecting approximately 1 in 4000 individuals worldwide [2]. RP is characterized by progressive bilateral degeneration of the rod and cone photoreceptors that leads to night blindness and progressive visual field defects [3]. Majority of RP cases are non-syndromic, whereas in 20–30 % cases are syndromic [4]. The most common form of syndromic RP is Usher syndrome (USH) and the general prevalence of USH approximately ranges from 1 to 4 in 25,000 individuals [5]. USH is characterized by congenital severe-to-profound sensorineural hearing loss with RP and rarely vestibular dysfunction. Depending on the severity, progression and age of onset of these features USH is divided into three clinical types: USH type 1 (USH1:MIM #276,901), USH type 2 (USH2: MIM#276,902) and USH type 3 (USH3:MIM#276,903), [6]. Each USH subtype is also genetically heterogeneous and several genes have been described for all three types.

The *USH2A* gene is located on chromosome 1q41 and has two alternatively spliced isoforms: a short isoform A and a long isoform B. A 5 kb short isoform is transcribed from 1to 21 exons of *USH2A* and translated into a putative 170 kDa extracellular secreted protein of 1,551 amino acids [7] that is expressed in both the retina and the inner ear [8]. A 15 kb long isoform is transcribed including additional 51 exons at the 3′ end of *USH2A* and translated into a 600-kDa transmembrane protein of 5202 amino acids [9, 10]. The dominant form of the USH2A protein, also known as usherin in photoreceptors is the 600-kDa polypeptide [9] that shows homology to both extracellular matrix proteins and receptors containing 10 laminin EGF-like domains, 35 fibronectine type-III (FN3) motifs, two laminin G domains and a PDZ-binding motif [9, 11]. This protein is expressed in adult human retina, specially localized to the photoreceptor cells and is required for the long-term maintenance of retinal photoreceptors and for the development of fetal cochlear hair cells [12]. The *USH2A* variants have been reported to cause recessive RP in 23 % cases [13], USH2 in 85 % cases [14]. In addition, the recessive *USH2A* variants have also been reported to cause non-syndromic hearing loss or deafness in few cases [15, 16]. According to our knowledge, no specific study has reported the association of *USH2A* variants with corneal diseases like Keratoconus (KC).However, studies have provided evidence for integration of *USH2A *into a protein network that is important in development and maintenance of the inner ear and retina [17–20]. In addition, mutations within *USH2A *affect its interaction with other proteins in network and may cause a broad spectrum of phenotypes in the inner ear and eye [18]. Therefore, to gain more insights into *USH2A* associated ocular diseases, we screened *USH2A *in Pakistani families with RP, USH2 and KC phenotypes in this study.

We identified a homozygous missense variants c.4029T > G, p.Asn1343Lys and c.7334 C > T, p. Ser2445Phe in the *USH2A *gene that segregated with KC and USH2 phenotypes in Pakistani families.

## Materials and methods

### Ethical approval and consent to participate

Approval was obtained from the Ethical Committee of Kohat University of Science and Technology (KUST), and the study was carried out in accordance with the Declaration of Helsinki.Informed written consent was obtained for participation in the study from families’ members and parents of the minor children. Families contained five living individuals and at least two living individuals with any type of rare inherited retinal or corneal disease with onset before age 20 years were eligible for the study. Following eligibility criteria, one non-syndromic RP, two USH2 and two KC families were recruited with help of ophthalmologists from the Khyber Pakhtunkhwa region of Pakistan. Demographic characteristics of recruited families are shown in Table 1.The phenotypically affected individuals in participated families underwent thorough ophthalmologic examinations for confirmation of clinical diagnosis of non-syndromic RP, USH and KC in them. The participated families were visited at the place of residence, pedigrees were drawn and disease associated features were recorded. Blood samples were collected from affected and normal individuals of both families. Blood samples were also collected from 100 ethnically matched unrelated healthy Pakistani individuals and were used as controls for allele frequencies and confirmation of disease associated variants.

**Table 1:** Demographic characteristics of investigated families

Families residential district	Disease	No. of living individuals		No. of affected individuals		Age of onset
		**Male**	**Female**	**Male**	**Female**	
Peshawar	RP	5	5	2	2	Childhood (around 10 years )
Karak	USH2	5	4	2	3	Congenital deafness, night blindness before10 years of age, pre-pubertal onset of RP
Kohat	USH2	4	2	2	1	Congenital deafness, night blindness after10 years of age, Onset of RP in early adolescence
Waziristan	KC	5	3	3	0	Early adolescence
Hangu	KC	8	5	3	2	Middle adolescence

### Detection of *USH2A *sequence variants

Genomic DNA from the blood samples was extracted using the GenElute™ Blood Genomic DNA kit (Sigma-Aldrich.com) according to the manufacturer’s protocol.

In this study direct Sanger sequencing and co-segregation analysis was performed to identify disease causing variants in the *USH2A* gene in all the recruited families. The coding region comprising of 2 to 72 exons and their intron–exon boundaries of the *USH2A* gene were amplified by polymerase chain reaction (PCR) in these families using primers reported previously [21], following standard conditions. The PCR amplified products were sequenced by the Macrogen sequencing service (Seoul, South Korea) in order to check the co-segregation of the variants with the disease phenotype in investigated families. The potential pathogenicity of each segregating sequence variant was determined using PolyPhen-2 (http://genetics.bwh.harvard.edu/pph2/), PROVEAN (http://provean.jcvi.org/index.php) and SIFT (http://sift.jcvi.org/) specialized prediction software. The conservation of a particular amino acid at a specific position was determined using Ensemble Wasabi viewer software (https://www.ensembl.org/index.html). To compare and correlate each *USH2A* gene variant with the disease phenotype, all reported variants were retrieved from HGMD (http://www.hgmd.cf.ac.uk/ac/search.php), OMIM (https://www.ncbi.nlm.nih.gov/omim/) and PubMed (https://www.ncbi.nlm.nih.gov/pubmed/) databases.

## Results

### Clinical characteristics

**Fig. 1:**
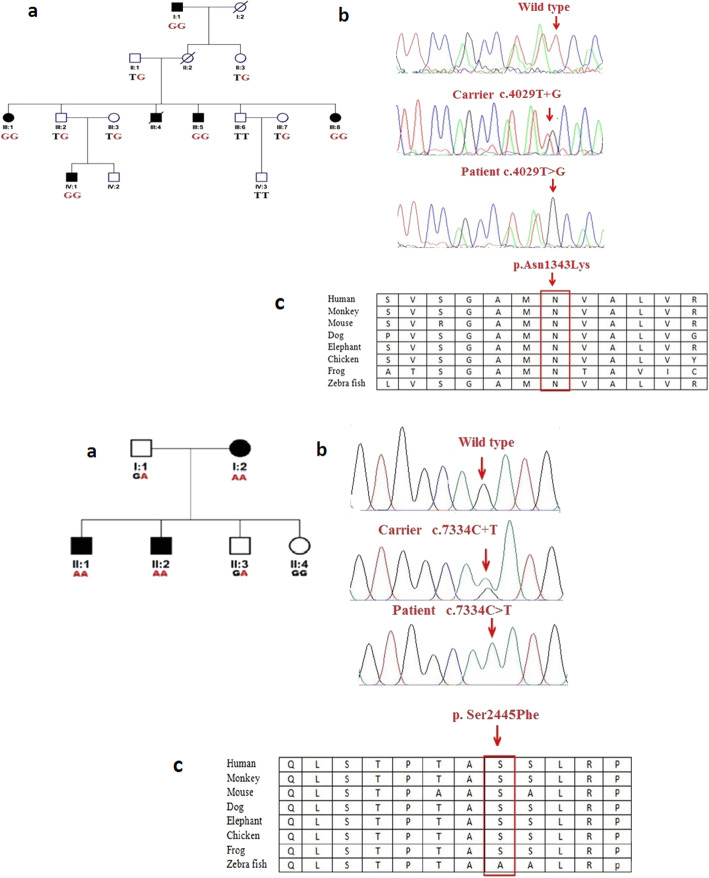
Family pedigrees, genotypes, and *USH2A *variants. (i) **a.** KC Family and (ii) a. USH2 family. In pedigrees, squares symbolize males and circles symbolize females. All filled circles and squares symbolize affected members, whereas clear circles and squares symbolize unaffected members. In addition, pedigrees affected by *USH2A *variants showing segregation of the altered alleles. (i) and (ii) **b.** Sequence chromatograms showing wild type and *USH2A* [c.4029T > G, p.Asn1343Lys (KC family), and, c.7334 C > T, p. Ser2445Phe (USH2 family)] variants. (i) and (ii) **c.** Multiple alignments of the partial amino acid sequences of *USH2A* in a variety of vertebrate and non-vertebrate species, show stringent conservation of Asparagine at position 1343 and Serine at position 2445

Disease causing variants in the *USH2A* gene were identified in a four-generation KC family and a two-generation USH2 family as shown in Fig. 1 (i) and (ii) a.Ophthalmology reports on affected members of both families confirmed clinical diagnosis of KC and USH2. The four affected individuals I: 1, III: 1, III: 5 and III: 8 were of 75, 37, 25 and 20 years of age respectively at the time of recruitment of KC family. KC occurred around 15 years of age in four affected individuals with symptoms include mild astigmatism, myopia, photophobia and eye itching and the degree of vision impairment increased with their increasing age. However, individuals younger than 10 years of age (IV: 1, IV: 2 and IV: 3) had no signs and symptoms of KC. The affected individuals in KC family were fitted with spectacle correction and two of them (III: 1 and III: 5) underwent the corneal cross linking surgery before age 21 years.

The three affected individuals II: 3, III: 1 and III: 2 were of 57, 29 and 27 years of age respectively at the time of recruitment of USH2 family. The affected individuals had bilateral, congenital, severe sensorineural hearing loss, normal vestibular function and symptoms of progressive RP. The affected individuals with USH2 experienced night blindness and progressive bilateral visual loss in early adolescence. Affected individuals (III: 1 and III: 2) were fitted with bilateral hearing aids and their affected mother (II:3) was blind and deaf at the time of recruitment of USH2 family.

### *USH2A* sequence variants

Mutation screening of the *USH2A* gene in KC family revealed a novel missense homozygous variant (NM_206933.4:c.4029T > G) in exon 17 condition in four affected individuals as well as in a phenotypically normal 6 years old child as shown in Fig. 1 (i) a and b. The variant c.4029T > G leads to a substitution of asparagine with lysine at the evolutionary conserved position 1343 (NP_996816.3:p.Asn1343Lys) according to the Ensemble databases as shown in Fig. 1(i) c. Insilco analysis of p.Asn1343Lys using PolyPhene-2, PROVEAN and SIFT predicted it as probably damaging or deleterious (Table 2).

**Table 2:** In silico analysis of the identified *USH2A* variants

Nucleotide change	Amino acid change	Exon	PolyPhen-2		PROVEAN		SIFT	
			**Prediction**	**Score**	**Prediction**	**Score**	**Prediction**	**Score**
c.4029G > C	p.Asn1343Lys	17	Probably damaging	1.000	Deleterious	-3.428	Damaging	0.01
c.7334 C > T	p. Ser2445Phe	19	Probably damaging	1.000	Deleterious	-3.008	Damaging	0.01

Direct sequencing analysis of the *USH2A* gene in USH2 family identified a missense homozygous variant (NM_206933.4: c.7334 C > T) in exon 19 in affected individuals and unaffected father and siblings were detected carriers for this variant as shown in Fig. 1 (ii) a and b. This single base pair change is predicted to result in substitution of serine with phenylalanine at evolutionary conserved residue position 2445 (p.Ser2445Phe) as shown in Fig. 1c (ii). This p.Asn1343Lys variant was also predicted probably damaging or deleterious or potentially disease causing by PolyPhene-2, PROVEAN and SIFT respectively (Table 2).

**Fig. 2:**
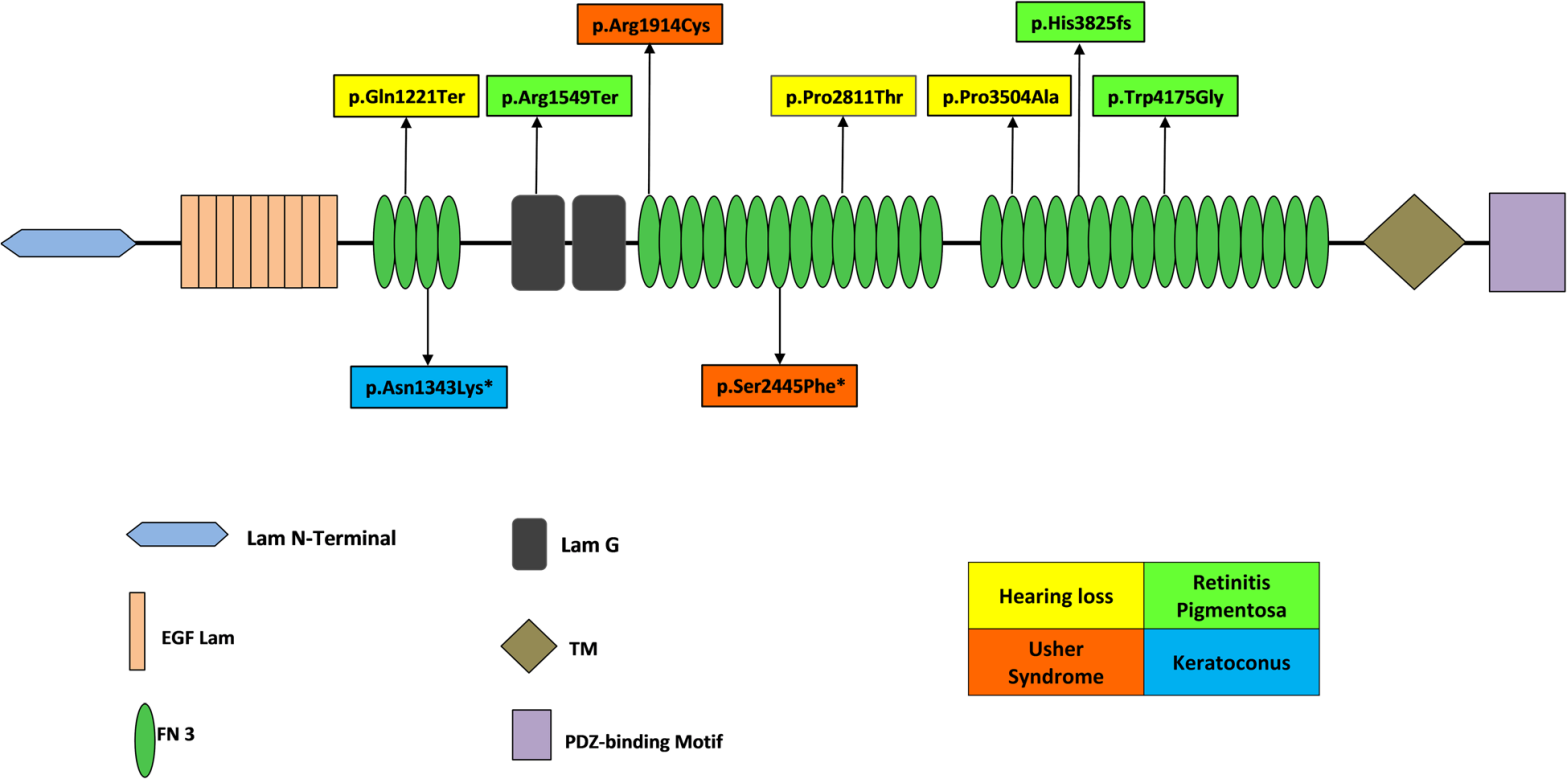
Schematic representation of domains of predicted protein product, highlighting the positions of all disease associated *USH2A* variants identified in Pakistani families to date. Discrete color pattern of variants shows type of phenotype

The identified *USH2A *variants in KC and USH2 families were found segregating in an expected autosomal recessive manner as shown in Fig. 1 (i) and (ii) a and were not detected in Pakistani unrelated healthy controls. A schematic representation of *USH2A *encoded usherin protein is presented to show spectrum of sequence variants identified in present study and previously reported in Pakistani families for various inherited ocular diseases in Fig. 2.

## Discussion

*USH2A* variants have been described to cause most commonly syndromic and non-syndromic forms of recessive IRD in different populations of world. High frequencies of *USH2A* variants have been reported in USH2 families of Jewish, Spanish, American, Scandinavian, Slovenian, and Italian, British origins [22–28]. In addition, *USH2A* variants have been identified to cause a substantial number of non-syndromic RP in families of Caucasian, Japanese, American and Chinese origins [13, 25, 29–33]. Importantly, *USH2A* variants display a wide phenotypic spectrum, therefore, phenotype-genotype correlation for the most prevalent *USH2A *variants may facilitate genetic counselling and improve the prognosis of affected individuals, as well as guide for patient-specific treatment options [26].

Available evidences suggest presence of *USH2A* recurrent variants in different populations as a result of founder effect. Of the 896 previously identified and previously reported *USH2A* variants in the Human Gene Mutation Database (HGMD), the p.Cys759Phe have been described the most prevalent pathogenic variant in the Spanish population and accounts for 4.5 % of the RP [34, 35] and 8.1 % of the USH2 cases [36], Similarly, a *USH2A* variant p.Trp3955Terwas detected in majority of Slovenian USH2 cases [26]. A deleterious USH2A variant p.Glu767fs have been found associated most commonly with USH2 [24, 37] and significantly with non-syndromic RP Caucasian patients [38]. A splice site founder variant (c.8559-2 A > G) of the *USH2A* was reported in majority of Chinese and Japanese USH2 patients [39, 40]. In addition, four *USH2A* variants c.239-240insGTAC, c.1000 C > T, c.2209 C > T, and c.12067-2 A > G account for 64 % USH2 in Jewish families of non-Ashkenazi descent [22]. A founder effect has been identified for a *USH2A* variant p.Cys1447fs that accounts for 55.6 % of the USH2 cases among Quebec French-Canadians [41]. However, great majority of reported *USH2A* variants are rare private mutations segregating in single families from different ethnic populations worldwide [42–45]. Involvement of *USH2A* private variants in causing profound hearing loss or deafness and IRDs in Pakistani families has been described in previous studies [16, 46–48]

Here, we report a novel missense private variant (c.4029T > G, p.Asn1343Lys) in the *USH2A* gene in a Pakistani family with KC phenotype. This variant was not found in population databases and was predicted deleterious or probably disease causing by in silico tools. The co-segregational analysis revealed that the four affected individuals and a clinically asymptomatic 6 years old individual in KC family were found homozygous for the identified variant. Importantly, this phenotype genotype difference in an individual could be attributed to the fact that the age of onset of disease symptoms was observed 15 years in investigated family. In this study we also report a missense variant (c.7334 C > T, p. Ser2445Phe) in the *USH2A* gene in another Pakistani family with USH2 phenotype. Noteworthy, the c.7334 C > T, p. Ser2445Phe variant was previously identified by Carss et al. [49] in a compound heterozygous form in a single individual with RP and present study provides evidence of this variant correlation with USH2 phenotype. It is a fact that both USH2 and non-syndromic RP are the most prevalent phenotypic variants of IRD caused by autosomal recessive *USH2A* variants, the findings of current investigation and previous one by Carss et al. [49] clearly demonstrate this fact and additionally, excluded the presence of a putative genetic modifier factor within the *USH2A* gene that may contribute in the development of two phenotypes.

Till date, mutation screening in Pakistani families revealed in total seven *USH2A* variants by previous researches [16, 46–48]. The *USH2A* variants described previously and in present study in Pakistani families thereby affecting functional residues absolutely conserved in the different domains of usherin protein. Among the two homozygous pathogenic USH2A variants identified in present study, a KC associated variant p.Asn1343Lys is located in the FN3 motif 2 and a USH2 associated variant p.Ser2445Phe is located in the FN3 motif 11. Previously reported homozygous pathogenic variants p.Gln1221Ter, p.Arg1914Cys, p.His3825fs, and p.Trp4175Gly in Pakistani families are located in the FN3 motifs 2,5,23 and 27 respectively. A compound heterozygous (p.Pro2811Thr and p. Pro3504Ala) variant identified in the *USH2A* gene in another Pakistani family was predicted to affect the FN3 motifs 14 and 20 respectively. All these variants reported in this study and previously affect highly conserved residues in the FN3 motifs across model organisms including human. The FN3 motifs have been shown to be involved in binding substrates thus highlighting the functional importance of the FN3 motifs in substrate selectivity. In addition, a nonsense *USH2A* variant p.Arg1549Ter identified previously in a Pakistani family [47], is located in the Laminin G-like 1 domain that result in production of truncated protein product and severe reduction in abundance of cellular transcript due to targeted degradation by nonsense-mediated decay.

Establishing the diagnosis for ocular diseases with greater clinical and genetic heterogeneity is quite difficult and challenging. This problem is usually more common in developing countries such as Pakistan, where people live in rural areas and generally have less access to healthcare and ophthalmic services. There is also limited availability of specific and expensive tests that are required for diagnostic investigations of ocular diseases. Mutation screening in *USH2A* in the current study enabled an accurate molecular diagnosis of *USH2A*-associated KC and USH2 to be established and has facilitated informed genetic counselling.

## Conclusions

This study revealed a novel genotype–phenotype correlation associated with *USH2A* (c.4029T > G, p.Asn1343Lys) variant as well as provided sufficient evidence to establish genotype–phenotype correlation of a *USH2A* variant (c.7334 C > T, p. Ser2445Phe) with USH2 phenotype. Analysis of genotype-phenotype correlation may increase our understanding of the diseases and may help in better management and counseling of affected individuals with *USH2A* variants and provides new targets for therapeutic approaches.

## Data Availability

All the data used to support the findings of this study are included within the article and are available on request from corresponding author.
